# Factors Associated with COVID-19-Related Stress among Female Primary Caregivers in Vulnerable Families in South Africa

**DOI:** 10.3390/ejihpe13020028

**Published:** 2023-02-02

**Authors:** Michelle Engelbrecht

**Affiliations:** Centre for Health Systems Research & Development, University of the Free State, Bloemfontein 9300, South Africa; engelmc@ufs.ac.za

**Keywords:** COVID-19, vulnerable, families, stress, finances, South Africa

## Abstract

Inequality in South Africa is deeply rooted, and COVID-19 glaringly brought inequalities between families to the forefront. This study aimed to investigate factors associated with the above average stress levels of female primary caregivers in vulnerable families during the COVID-19 pandemic. A cross-sectional survey was undertaken among vulnerable families from October 2021 to February 2022. Above average scores were reported by approximately half of the respondents regarding stress from children/partners and stress related to financial issues. Fear of COVID-19, feeling depressed, COVID-19′s impact on daily life, lower education levels, being in a relationship and living together, and perceiving an increase in domestic violence were statistically significantly associated with above average stress from children/partners. Age, impact of COVID-19 on daily life, being in a relationship and living with a husband/partner, and running out of food during the past 30 days were statistically significantly associated with above average stress concerning finances. These results provide new insights that can assist policy makers and practitioners in supporting low-income families during times of crisis. Support should not just focus on practical aspects, such as the provision of food, but equally importantly, on emotional support and protection for female primary caregivers and their families. Future research should delve more deeply into causes of COVID-19-related stress in vulnerable families.

## 1. Introduction

COVID-19 is more than a global health crisis; it has disrupted all aspects of everyday life from economic stability to the psychosocial well-being of individuals [1,2,3,4]. In late 2019, the World Health Organization’s (WHO) Country Office in the People’s Republic of China was alerted to a media statement on cases of ‘viral pneumonia’ in Wuhan, capital of the Hubei Province. It was subsequently reported that the outbreak in Wuhan was caused by the 2019 Novel Coronavirus (2019-nCoV). By early March 2020, more than 118,000 cases were reported across 114 countries, and the WHO declared COVID-19 a pandemic [5]. In order to curb the spread of the disease, countries worldwide implemented lockdown measures, and by early April 2020, more than one-third of the world’s population was under some form of movement restriction [6]. Restrictions implemented in South Africa were among the most stringent [7]. Initially, only essential workers were permitted to go to work; schools were closed; individuals were encouraged to work from home, and could only leave to purchase food and seek medical assistance. Restrictive measures led to job losses, poverty, and isolation from protective social networks, with families stripped of the resources necessary to care for their children [8].

Inequality in South Africa is deeply rooted, exhibiting the highest levels of inequality in the world, with almost one-fifth of the population living in extreme poverty [9]. Gross social and economic inequalities have meant that some groups are more severely impacted by COVID-19 than others. In particular, inequalities between families have been brought glaringly to the forefront [10], where practicalities such as overcrowding, cramped living conditions, lack of running water in homes, etc., made it almost impossible for low-income families to comply with lockdown regulations [11]. In short, COVID-19 changed family life, with those living in low-income households experiencing the brunt of the burden [3,12,13].

COVID-19-related loss of income [3,14,15,16], attributed to, among other factors, loss of jobs, closure of businesses, disruption of farming activities, increasing input prices, decreasing output prices, increasing food prices, and illness or death of an income earner, is a major challenge faced by families. Research across low-income African countries found that families attempt to cope with the loss of income by using savings, selling assets, reducing food consumption, and receiving assistance from family or the government [16]. To help compensate for income lost due to lockdown restrictions, the South African government introduced social relief measures, including a special COVID-19 ZAR 350 (USD 20.67) social relief of distress grant [7], which resulted in an increase in households receiving at least one social grant from 45.5% in 2019 to 52.4% in 2020 [17].

Research also shows a significant relationship between economic hardship related to COVID-19 and psychosocial wellbeing [15,18,19,20]. Mandatory stay-at-home orders disrupted families’ daily routines, restricted family gatherings and the opportunity to socialize, increased family conflicts and domestic violence, and caused increased stress for parents [14,20,21,22]. International research highlights factors associated with the increased level of stress among parents and include: changes in daily routine, worry/anxiety about COVID-19, lack of money, and lack of food [21]. The impact on family life was further aggravated by the closure of schools, which led to disruptions in education and posed severe challenges for children from lower income households, who do not have access to the necessary electronic devices or internet to participate in online educational activities [8,14,16]. According to a joint UNESCO, UNICEF, and World Bank report [23], the pandemic caused the largest disruption to education in history, with over 1.6 billion students forced to stay home at some point. This resulted in serious learning losses, with some estimates suggesting that students may have lost as much as 50% of their learning gains due to the pandemic, with younger and more marginalized children often showing the greatest losses. Additionally, the pandemic had a psychological impact on students, with many reporting increased levels of stress and anxiety. Several risk factors, such as living in rural areas and low family socioeconomic status, have been found to be associated with worse mental health outcomes [24]. School closures also impacted food security, as children from lower-income groups often receive a meal at school [8,25].

Given the aforementioned challenges imposed by the pandemic and the subsequent measures to control the spread of the disease, this paper focuses on the COVID-19-related family stress of vulnerable families. There are various types of families in South Africa (e.g., nuclear families, extended families, single parent families, families headed by teenagers or grandparents, etc.) [26]; consequently, it is not surprising that COVID-19 impacted different types of families dissimilarly. Families headed by women are especially vulnerable to the economic impact of COVID-19 [27], with more women losing their jobs or working fewer hours than men. Women were also required to spend more time providing childcare as a result of school and day-care closures [28]. In South Africa, approximately two out of five mothers are single parents, and three out of five children are from families where the father is absent. These families are mainly from previously disadvantaged communities that prior to COVID-19, were already characterized by low levels of education and high levels of unemployment. It is important to keep in mind that female-headed families are not necessarily single-parent families; the father may be working away from home, or the family may be part of a multi-generational or extended family [27]. Therefore, this study aimed to investigate factors associated with above average stress levels of primary female caregivers in vulnerable families residing in a metropolitan area of South Africa during COVID-19.

## 2. Materials and Methods

### 2.1. Design and Sample

A cross-sectional survey was undertaken among vulnerable families residing in an urban metropolitan area. Respondents were recruited in collaboration with three civil society organizations providing welfare services in the study area. The inclusion criteria were a female primary caregiver (i.e., the mother), older than 18 years, identified as vulnerable by any of the civil society organizations assisting with recruitment, and currently living with at least one biological/adopted child younger than 18 years of age, and who may/may not be residing with a partner. A total of 275 female primary caregivers participated in the study. The average age of the respondents was 36.25 years (SD 8.525). Half of the respondents (50.7%) had a secondary school education, while around one-third (32.5%) had completed Grade 12. Approximately half of the respondents were not married, lived without a partner (49.5%), and were the head of their households (49.6%). The majority of respondents (84.4%) had children who attended school. The demographic characteristics of the respondents are presented in Table 1.

### 2.2. Data Collection

Community care workers from the three civil society organizations recruited female primary caregivers. Trained fieldworkers, who were fluent in Sesotho and English, obtained written informed consent from the respondents. The fieldworkers read the questions directly from the questionnaire, which was available electronically on a tablet. The questions were closed-ended, with response options provided. The option “Other, please specify” was available, where necessary. The structured interview was conducted at a time and place most convenient for the respondents. The questionnaires were available in the most frequently spoken local languages, Sesotho and English. All COVID-19 infection control protocols, including social distancing, wearing masks, and sanitizing, were adhered to during data collection.

The questionnaire was comprised of four sections, which collected demographic and socio-economic information, as well as responses to the Fear of COVID-19 Scale [29] and the COVID-19 Family Stressor Scale (CoFaSS) [30]. The Fear of COVID-19 Scale is a seven-item scale assessing fear of COVID-19 among the general public. Respondents are asked to rate their agreement with each statement on a 5-point scale from “1—Strongly Disagree” to “5—Strongly Agree.” Higher scores indicate greater fear. The scale has been found to be reliable and valid in assessing fear of COVID-19 among the general population [29] and has been widely used, as indicated in a systematic review and meta-analysis of 44 articles using the Fear of COVID-19 Scale [31]. The one-factor model and reliability of the scale has been supported in subsequent studies [32,33,34], including studies conducted in South Africa [35,36].

The CoFaSS comprises three sub-scales and 16 items measuring income stress, family stress, and chaos stress. Respondents are asked “Since the COVID-19 disruption, have any of the following changes occurred in your household?” with responses indicated on a three-point Likert scale—“Not True” (1), “Somewhat True” (2), and “Very True” (3). The CoFaSS has been validated for use with White/European male and female caregivers, with the developers recommending that additional validation efforts are required across diverse ethnic/racial and socioeconomic groups [30].

### 2.3. Data Analysis

The data was transferred from the electronic questionnaire into Excel and then exported to SPSS. Data that was recorded under the “Other, please specify” option were coded and quantified. Data was analyzed in IBM SPSS version 28 (IBM Corp., New York, NY, USA) [37] and Python [38]. The data was described using frequency counts and percentages for categorical variables and means and standard deviations for continuous variables. As the CoFaSS has not been validated for our particular respondent group, exploratory factor analysis, with varimax rotation, was conducted to determine the underlying factor structure. Bartlett’s test of sphericity and the Kaiser–Meyer–Olkin (KMO) test were used to check the suitability of the data for exploratory factor analysis. Cronbach’s alpha was used to determine the internal consistency of the CoFaSS. Binary logistic regression analyses were performed to ascertain the factors associated with stress from financial issues and stress from children/partners. Univariate analysis was used to identify variables for the inclusion in the binary logistic regression analyses. In this regard, univariate analysis was performed to determine the effects of age, fear of COVID-19, feeling depressed (Yes/No), impact of COVID-19 on daily life (large impact/small impact), education (Grades 1–11/Grade 12+), relationship status (in a relationship and living with partner/in a relationship but not living with partner, living without a partner), employment status (employed/unemployed), and having run out of food in the month prior to the fieldwork (Yes/No) on financial stress. Similarly, a univariate analysis was also conducted to determine the effects of age, fear of COVID-19, feeling depressed, impact of COVID-19 on daily life, education, relationship status, observing domestic violence in the community (Yes/No), and employment status on above average stress from children and/or partner. Only significant variables were included in the final models. The outcome variables were dichotomized to reflect average/below stress and above average stress, based on the mean scores for these subscales. The Hosmer–Lemeshow test, which is similar to a χ2 goodness of fit test, was used to assess the goodness of fit of the models. Odds ratios (ORs), together with their corresponding 95% confidence intervals (CIs), were estimated. The significance level considered for this study was 0.05.

## 3. Results

### 3.1. Biographic and Socio-Economic Information

The respondents relied heavily on social grants, with 57.1% of the women receiving their own social grant, which was mainly the COVID-19 grant of ZAR 350-00 (91.5%). In addition, most women (92.4%) also indicated that their children received a child support grant. Approximately one-quarter of the respondents (24.7%) were employed. Of the women who were employed prior to COVID-19, and who were no longer employed at the time of the fieldwork, 30.7% reported losing their jobs due to COVID-19. Almost half of the respondents experienced a decrease in income since COVID-19 (46.9%) and were worried about not having enough food to eat (56.6%). Approximately three-quarters of the women (73.8%) tried to cope with the impact of COVID-19 by reducing food consumption, while 42.5% borrowed money from families or friends. Slightly more than one-third of the respondents reported increased domestic violence in the community since COVID-19 (34.8%), while 35.9% were of the opinion that there had not been a change in levels of domestic violence in the community (Table 2).

### 3.2. Fear of COVID-19

Out of a maximum of 35, the mean score on the Fear of COVID-19 scale was 22.95 (SD 7.1842). The highest mean scores for individual items on the scale were from, “I am very afraid of the COVID-19 virus” (mean 4.07; SD 1.403) and “I feel uncomfortable thinking about the COVID-19 virus” (mean 3.50; SD 1.394) (Table 3).

The Fear of COVID-19 Scale had a Cronbach’s alpha of 0.8, suggesting that the scale was a reliable measure of fear for the current population. In addition to fear of COVID-19, three out of five respondents (60.4%) indicated that they had felt depressed during the past 30 days.

### 3.3. COVID-19 Family Stress

Descriptive statistics for items on the original Family Stress Scale [30] are provided in Table 4. Items with the highest average stress scores included: concern about providing for family (mean 2.35; SD 0.723), experiencing a significant decrease in household income (mean 2.32; SD 0.765), and not being able to access essential supplies (mean 2.11; SD 0.736).

An exploratory factor analysis was conducted on a 16-item Family Stress Scale [30], measuring stress from disruptions related to the COVID-19 pandemic regarding finances, basic needs, personal and family welfare, career/education, household responsibilities, and family relationships. An orthogonal rotation (varimax) was used. The Kaiser–Meyer–Olkin measure verified the sampling adequacy for the analysis, with KMO = 0.81. Bartlett’s test of sphericity X^2^ = 1186, *p* < 0.001, indicated that the correlations between items were sufficiently large for exploratory factor analysis. An initial analysis was conducted to obtain eigenvalues for each factor in the data. Four factors showed eigenvalues over Kaiser’s criterion of 1; therefore, four factors were retained for the analysis. After running the analysis with four factors, only one item loaded on the fourth factor and the analysis was run for three factors. Two items (6 and 14) were dropped, as they did not load above 0.32 on any factors, while a third item (15) was dropped, as it loaded highly on two factors. In addition, items 5 and 8 were dropped, as they did not naturally fit in with any of the identified factors. The three-factor structure explained 44% of the variance. Table 5 shows the factor loadings after rotation. The items that cluster on the same factors suggest that Factor 1 is concerned with stress from immediate family (i.e., children and partner), Factor 2 assesses financial stressors, and Factor 3 includes stressors relating to the family in general.

The new factor structure was slightly different from the original CoFaSS [30]. It still includes financial stress; however, family stress was dichotomized into children/partners stress (focused on child management, educational materials for children, increased conflict with partner, and anxiety for loved ones) and stress from family in general (addressing altercations and emotional withdrawal). Items related to chaos stress were dropped, which could be due to the dire socio-economic circumstances of the respondents, where it is almost impossible for people to withdraw from social interactions in a single home, work remotely, buy large quantities of supplies to avoid regular visits to the shops, or drive alone in a car to purchase supplies [11]. Furthermore, the distinction between stress from children/partners versus stress from “family” could be explained by the criteria used to sample our female primary caregivers, which emphasised the “nuclear family,” while the extended family (i.e., a multigenerational family that may or may not share the same household) in Black South African culture is particularly important and valued [26,39].

All three subscales had a Cronbach’s alpha of 0.7, suggesting that the subscales were reliable measures of stress for the current population.

The mean (average) scores for the revised Family Stress subscales are as follows: stress for partner/child (mean 5.93; SD 1.997), financial stressors (mean 10.43; SD 2.670), and stress from extended family (mean 3.29; SD 1.098) are depicted in Table 6. Above average scores were reported by 54.2% of respondents for stress from children/partners, 50.5% for stress from extended family, and 49.8% for stress related to financial issues.

### 3.4. Factors Associated with COVID-19 Family Stress

Logistic regression was used to determine the factors associated with above average stress from children and/or partner (Table 7) and above average financial stress (Table 8).

Individual variables that were found to be statistically significantly associated with above average stress from children/partners were: fear of COVID-19 (OR: 1.047; *p* = 0.010), feeling depressed (OR: 2.011; *p* = 0.004), COVID-19 impact on daily life (OR: 1.769; *p* = 0.032); lower education levels (OR: 1.848; *p* = 0.020); being in a relationship and living together (OR: 3.234; *p* < 0.001); and perceiving an increase in domestic violence in the community (OR: 2.456; *p* = 0.004). These six variables were included in the adjusted model. No outliers, high-leverage values, or influential points were identified in the data. The model was statistically significant, implying that the predictors, as a set, reliably distinguished between female primary caregivers who experienced lower levels of stress related to children and family and those who experienced above average stress levels, X^2^(8) = 47.401; *p* < 0.001. The Hosmer and Lemeshow test also indicated that the model was a good fit, X^2^(8) = 4.754, *p* > 0.05). The model explained 21.5% of the variance (Nagelkerke R^2^) in the tendency to experience above average stress from children/partners and correctly classified 66.8% of the cases. The six variables were all found to be statistically significantly associated with above average stress from children/partners (*p* < 0.05). The higher the score on the Fear of COVID-19 scale, the more likely the respondents were to also have above average stress from children and/or partners (OR: 1.040; *p* = 0.039). Compared to respondents who did not feel depressed, those who did feel depressed were 1.8 times more likely to also experience above average stress from children and/or partners (OR: 1.774; *p* = 0.038). Respondents who indicated that COVID-19 had a large impact on their daily lives (OR: 2.023; *p* = 0.017) and who perceived an increase in domestic violence in their community (OR: 2.091; *p* = 0.030) were twice as likely to also have above average stress from children and/or partners. Respondents who were in a relationship and living with their husband/partner were approximately 3 times more likely to also experience above average stress from children and/or partners compared to persons who were not in a relationship (OR: 2.895; *p* = 0.001). Respondents who had not completed their schooling, were 2.4 times more likely to also experience stress from their children and/or partner (OR = 2.372; *p* = 0.003).

Individual variables associated with above average stress from finances and found to be statistically significant were: age (OR: 1.044; *p* = 0.004); feeling depressed (OR: 1.746; *p* = 0.023); impact of COVID-19 on daily life (OR: 2.140; *p* = 0.004); being in a relationship and living with a husband/partner (OR: 2.184; *p* = 0.007); being unemployed (OR: 1.843; *p* = 0.033); and family (i.e., self, husband/partner and children), and sometimes (OR: 2.896; *p* = 0.004) or often (OR: 5.826; *p* < 0.001) not having food to eat during the past 30 days. These five variables were included in the adjusted model. No outliers, high-leverage values, or influential points were identified in the data. The model was statistically significant, implying that the predictors, as a set, reliably distinguished between female primary caregivers who experienced lower levels of stress related to finances compared to those who experienced above average stress levels, X^2^(8) = 52.194, *p* < 0.001. The Hosmer and Lemeshow test also indicated that the model was a good fit, X^2^(8) = 6.153, *p* > 0.05). The model explained 23.2% of the variance (Nagelkerke R2) in the tendency to experience above average stress from children/partners and correctly classified 67.8% of the cases. Age, impact of COVID-19 on daily life, being in a relationship and living with husband/partner, and running out of food during the past 30 days were found to be statistically significantly associated with above average stress from finances. Older persons were more likely to experience above average financial stress (OR: 1.041; *p* = 0.014) than younger persons. Respondents who felt that COVID-19 had a large impact on their daily lives (OR: 1.969; *p* = 0.019) were twice as likely to experience above average financial stress. Women who were in a relationship and living with their husband/partner were two times more likely to experience above average financial stress compared to those women who were not in a relationship and living without a partner (OR: 2.146; *p* = 0.017). Families (i.e., the respondent and their children and/or partners) that sometimes (OR: 3.387; *p* = 0.002) or often (OR: 5.826; *p* < 0.001) ran out of food during the past month were respectively three and six times more likely to also experience above average financial stress compared to families who had not run out of food.

## 4. Discussion

This study aimed to investigate factors associated with above average stress levels of female primary caregivers in vulnerable families during COVID-19. Approximately half of the respondents reported above average stress levels, particularly stress from children/partners and financial constraints. Given the dire socio-economic consequences of COVID-19 [40,41], as well as the inherent difficulties that families from lower socio-economic groups were confronted with when complying with lockdown restrictions (i.e., overcrowded living conditions and the lack of access to basic services, such as water and sanitation within the home) [42] and the protracted closure of schools [22], higher stress levels are not unexpected. Factors that predicted above average stress from children and/or partners during COVID-19 included emotional reactions such as fear of the disease, feeling depressed, and perceiving that COVID-19 had a substantial impact on daily life, as well as socio-demographic factors including age, level of education being in a relationship and living together, and perceiving an increase in domestic violence in the community. Similarly, socio-demographic factors, including age, being in a relationship and living with a husband/partner, running out of food during the past 30 days, and as perceiving COVID-19 to have impacted daily life, were associated with above average financial stress.

The rapid spread of the disease and lockdown restrictions, together with their associated impact, has resulted in different psychological effects on individuals [15,18,19,20,43,44]. Similar to the current study, other research also reports on the significant association between fear of COVID-19, depression, and stress related to COVID-19 [44]. The mean score on the Fear of COVID-19 Scale suggests that the respondents experienced higher levels of fear. A systematic review of studies that utilized the Fear of COVID-19 Scale identified a pooled mean score of 20.67 for women, which was slightly lower than that in the current study, in which the mean was 22.95. Excessively high levels of fear can have a negative influence on psychological wellbeing, while normal to moderate levels of fear are needed to reduce the risk of contracting COVID-19 [31]. The higher levels of fear of COVID-19 reported by the respondents seem to go hand-in-hand with feeling depressed (three in five respondents indicated feeling depressed), which was also associated with above average stress from children/partners.

The association between level of education and above average stress from children/partners could be explained by the demands of home-schooling children as a result of school closures during lockdown restrictions. The digital divide in South Africa meant that learners from poorer families had little access to online learning, while parents, many of whom lack educational capital, were forced to take on teaching responsibilities [7]. It is possible that less educated primary caregivers might have felt less competent to assist their children with schoolwork and as a result, they experienced more stress than caregivers with higher education levels. Research among a sample of Italian parents revealed a link between parental stress and home-schooling [45]. Furthermore, Pressman et al. [46] identified a link between caregiver’s perceived ability to help their children with homework and increased family-related stress.

The closure of schools during lockdown restrictions also impacted food security, as children were no longer able to access meals at school. Prior to lockdown, more than nine million children at schools in the poorest three quintiles received daily meals through the National School Nutrition Program, and approximately 2.5 million pre-schoolers attending early childhood development programs were also provided with a meal. While government departments and NGOs attempted to address this issue during lockdown, they could not fill the gap left by the suspension of the school feeding program [8]. This challenge would appear to be related to the finding that the majority of respondents reported that their families had run out of food in the month prior to the fieldwork, and that this was the main factor contributing to financial stress.

Respondents who lived with their partners were three times more likely to experience stress from children and/or partners than those who lived alone and two times more likely to experience financial stress, suggesting that confinement during lockdown may have increased conflicts in the home. Statistics have indicated that lockdown restrictions implemented in South Africa placed vulnerable groups at an increased risk for violence [14,47]. During the first week of lockdown in March 2021, the South African Police Service received 2 300 calls for help related to gender-based violence [48]. Research has found that persons who live with partners are more likely to experience financial stress, which is also associated with intimate partner violence [49]. This could be related to the finding that respondents who perceived an increase in domestic violence in their community were also more likely to experience above average stress from children/partners.

While there is existing research on the wellbeing of families during COVID-19 in South Africa [22,50,51], it has also been noted that there is a need for more studies to identify the influence of COVID-19 on the diverse forms of families in this country [22]. Therefore, the value of the current study is that it is one of the first to focus on different sources of COVID-19-related stress on vulnerable families. However, as with all research, this study also has limitations. Firstly, the cross-sectional nature of the study did not allow for interpretation of causality. Further research is required to delve more deeply into the causes of stress experienced by vulnerable families during COVID-19. Secondly, due to difficulties in accessing fathers, only female primary care givers were included in the study. It would be beneficial for future research to also include the perspectives of fathers. Thirdly, as fieldworkers administered the questionnaires, there is a possibility of response bias, as respondents may have been more inclined to provide answers that would paint them in a favorable light. Fourthly, the questionnaires were mainly comprised of questions with structured response categories, while the inclusion of more open-ended questions would have afforded the respondents the opportunity to elaborate on their answers. Finally, experiencing depression was measured with a single item, and not a scale. It is recommended that future studies use a validated scale to measure this complex construct.

## 5. Conclusions

COVID-19 placed an additional burden on already thinly stretched low-income families living in poor socio-economic conditions. Above average stress levels were associated with factors including: age, level of education, being in a relationship and living together, fear of the disease, feeling depressed, perceiving that COVID-19 had a substantial impact on daily life, perceiving an increase in domestic violence in the community, and running out of food during the past 30 days. The results from this study can help us to better understand the socio-economic impact of COVID-19 on vulnerable families and provides new insights that can assist policy makers and practitioners to support low-income families during times of crisis. In this regard, the Department of Social Development, as well as NGOs that provide emotional support services, are key to assist vulnerable families during times of crisis. A crucial message is that support should not just focus on practical aspects, such as the provision of food, but should equally importantly focus on emotional support and protection for female primary caregivers and their families. Future research should delve more deeply into causes of COVID-19-related stress in vulnerable families.

## Figures and Tables

**Table 1:** Demographic characteristics.

Variables	Responses	*n*	%
Marital status and living arrangements (N = 275)	Married, living together	48	17.5
	Married, not living together	16	5.8
	Not married and living without a partner	136	49.5
	Living with partner	32	11.6
	Partner lives elsewhere	43	15.6
Education (N = 274)	No schooling	1	0.4
	Primary school (Grades 1–7)	41	15.0
	High school (Grades 8–11)	139	50.7
	Grade 12 (completed schooling)	89	32.5
	Degree/Diploma	4	1.4
Head of the family (N = 274)	Head of the family	136	49.6
	Not the head of the family	138	50.4
Living with children (N = 275)	Children younger than 6 years	43	15.6
	Children 6–17 years	217	79.2
	Children 18 years and older	61	22.2
Children attend school (N = 275)	Children do not attend school	43	15.6
	Children attend school	232	84.4

**Table 2:** Socio-economic characteristics.

Variables	Responses	*n*	%
Domestic violence in the community since COVID-19 (N = 273)	Decrease in domestic violence	80	29.3
	Unchanged levels of domestic violence	98	35.9
	Increase in domestic violence	95	34.8
Social grants (N = 275)	Social grants not received	157	57.1
	Social grants received	118	42.9
Categories of social grants received (N = 118)	COVID-19 Grant (ZAR 350)	108	91.5
	Disability grant	7	5.9
	Older persons grant	2	1.7
	Social relief of distress	1	0.8
Child support grant (N = 275)	Child support grant received	254	92.4
	Child support grant not received	21	7.6
Employment status (N = 275)	Employed prior to COVID-19 outbreak	116	42.2
	Currently employed	68	24.7
Job loss due to COVID-19 (N = 163)	Job lost due to COVID-19	50	30.7
	Job not lost due to COVID-19	113	69.3
Change in income due to COVID-19	No income	60	21.8
	Income increased	20	7.3
	Income remained the same	66	24.0
	Income reduced	129	46.9
Run out of food in the last 30 days	No, never	26	9.5
	Yes, often	118	42.9
	Yes, sometimes	99	36.0
	Yes, seldom	32	11.6
Worried about not having enough to eat in the next week (N = 274)	Not worried at all	9	3.3
	Not too worried	27	9.8
	Somewhat worried	83	30.3
	Very worried	155	56.6
Financial coping strategies since COVID-19 (N = 275)	Borrowed money from friends or family	117	42.5
	Received non-monetary assistance from friends or family	93	33.8
	Borrowed money from an informal money lender	99	36.0
	Delayed making repayments	83	30.2
	Reduced food consumption	203	73.8

**Table 3:** Descriptive statistics of the Fear of COVID-19 Scale.

Scales	Minimum	Maximum	Mean	SD
I am very afraid of the COVID-19 virus.	1	5	4.07	1.403
I feel uncomfortable thinking about the COVID-19 virus.	1	5	3.50	1.394
I don’t work when I think about COVID-19 virus.	1	5	2.82	1.420
I am afraid to lose my life due to COVID-19 virus.	1	5	4.11	1.424
When I watch news and stories about the COVID-19 virus on social media, I am tense or anxious.	1	5	3.32	1.362
I can’t sleep because I am worried about infection with the COVID-19 virus.	1	5	2.68	1.347
My heart speeds or beats when I think about infection with COVID-19 virus.	1	5	2.45	1.244

**Table 4:** Descriptive statistics of the Family Stress Scale.

Scales	Minimum	Maximum	Mean	SD
Q1 Significant decrease (over 10%) in household income	1	4	2.32	0.765
Q2 Gone into financial debt	1	4	1.92	0.848
Q3 Job disruption or loss (myself or my partner)	1	4	1.73	0.892
Q4 Could not access essential supplies (e.g., sanitizer, soap, toilet paper, etc.)	1	4	2.11	0.736
Q5 Overwhelmed by the amount of COVID-19 news coverage	1	4	1.82	0.742
Q6 Applied for employment insurance or government assistance	1	4	1.44	0.689
Q7 Became concerned about providing for my family	1	4	2.35	0.723
Q8 Became stressed by crowded grocery stores and shopping centers	1	4	1.89	0.776
Q9 Experienced increased altercations with family members	1	4	1.68	0.652
Q10 Experienced increased emotional withdrawal from family members	1	4	1.61	0.603
Q11 Children have become harder to manage	1	4	1.51	0.738
Q12 Inability to access educational materials for children	1	4	1.61	0.715
Q13 More relationship conflicts with my partner (if I am in a relationship)	1	4	1.41	0.692
Q14 Difficulty developing a new family and/or personal routine	1	4	1.51	0.595
Q15 Felt crowded in my living space	1	4	1.51	0.637
Q16 Significant anxiety/panic about danger to myself or loved ones	1	4	1.40	0.592

**Table 5:** Exploratory factor analysis of the Family Stress Scale.

Item	Factor 1	Factor 2	Factor 3
Q1 Significant decrease (over 10%) in household income	0.083	**0.541**	0.055
Q2 Gone into financial debt	0.193	**0.471**	0.0535
Q3 Job disruption or loss (myself or my partner)	0.102	**0.564**	0.141
Q4 Could not access essential supplies (e.g., sanitizer, soap, toilet paper, etc.)	0.172	**0.635**	0.201
Q7 Became concerned about providing for my family	0.226	**0.504**	0.121
Q9 Experienced increased altercations with family members	0.184	0.229	**0.701**
Q10 Experienced increased emotional withdrawal from family members	0.222	0.136	**0.750**
Q11 Children have become harder to manage	**0.799**	0.142	0.117
Q12 Inability to access educational materials for children	**0.679**	0.203	0.152
Q13 More relationship conflicts with my partner (if I am in a relationship)	**0.460**	0.143	0.140
Q16 Significant anxiety/panic about danger to myself or loved ones	**0.590**	0.217	0.143

**Table 6:** Descriptive statistics for the revised Family Stress Scale.

Scales (N = 273)	Minimum	Maximum	Mean	SD
COVID-19 Stress—Children/partners	4	12	5.93	1.997
COVID-19 Stress—Financial	5	15	10.43	2.670
COVID-19 Stress—Family	2	6	3.29	1.098

**Table 7:** Factors associated with above average stress from children and/or partners.

Variable	Unadjusted Odds Ratio (95% CI)	Adjusted Odds Ratio (95% CI)
Age	1.016 (0.988–1.045)	-
Fear of COVID-19	1.047 (1.011–1.083) *	1.040 (1.002–1.080) *
Depressed		
- No - Yes	1 2.011 (1.254–3.226) *	1 1.774 (1.034–3.044) *
Impact of COVID-19 on daily life		
- Small impact - Large impact	1 1.769 (1.050–2.979) *	1 2.023 (1.132–3.617) *
Education		
- Completed school - Not completed school	1 1.848 (1.103–3.097) *	1 2.373 (1.334–4.223) *
Relationship		
- Not in a relationship - In a relationship and living together - In a relationship and not living together	1 3.234 (1.813–5.769) * 1.345 (0.719–2.517)	1 2.895 (1.536–5.457) * 1.409 (0.715–2.776)
Domestic violence in the community		
- Improved - Worse - Same	1 2.456 (1.332–4.529) * 1.028 (0.558–1.893)	1 2.091 (1.074–4.072) * 1.007 (0.524–1.937)
Employment		
- Employed - Unemployed	1 1.240 (0.710–2.164)	-

* *p* < 0.05.

**Table 8:** Factors associated with above average financial stress.

Variable	Unadjusted Odds Ratio (95% CI)	Adjusted Odds Ratio (95% CI)
Age	1.044 (1.014–1.075) *	1.041 (1.008–1.075) *
Fear of COVID-19	1.030 (0.996–1.066)	-
Depressed		
- No - Yes	1 1.764 (1.080–2.882) *	1 1.382 (0.807–2.366)
Impact of COVID-19 on daily life		
- Small impact - Large impact	1 2.140 (1.272–3.600) *	1 1.969 (1.116–3.475) *
Education		
- Completed school - Not completed school	1 0.829 (0.501–1.373)	-
Relationship		
- Not in a relationship - In a relationship, living together - In a relationship, not living together	1 2.184 (1.236–3.858) * 1.103 (0.595–2.045)	1 2.146 (1.144–4.025) * 1.094 (0.554–2.159)
Employment		
- Employed - Unemployed	1 1.843 (1.050–3.234) *	1 1.847 (0.981–3.479)
Family (i.e., children and husband/partner) ran out of food in past 30 days		
- Never - Sometimes - Often	1 2.896 (1.409–5.952) * 5.826 (2.861–11.865) *	1 3.387 (1.579–7.266) * 5.164 (2.456–10.858) *

* *p* < 0.05.

## Data Availability

Data supporting the reported results can be requested from the first author.
